# DNA Damage Response Evaluation Provides Novel Insights for Personalized Immunotherapy in Glioma

**DOI:** 10.3389/fimmu.2022.875648

**Published:** 2022-05-26

**Authors:** Mu Chen, Bingsong Huang, Lei Zhu, Qi Wang, Ying Pang, Meng Cheng, Hao Lian, Min Liu, Kaijun Zhao, Siyi Xu, Jing Zhang, Chunlong Zhong

**Affiliations:** ^1^Department of Neurosurgery, Shanghai East Hospital, School of Medicine, Tongji University, Shanghai, China; ^2^Institute for Advanced Study, Tongji University, Shanghai, China

**Keywords:** DNA damage response, immunotherapy, glioma, tumor microenvironment, immune checkpoint blockade, DNA repair

## Abstract

**Background:**

DNA damage response (DDR) proficiency is the principal mechanism of temozolomide (TMZ) resistance in glioma. Accumulating evidence has also suggested the determining role of DDR in anticancer immunity. We propose that a comprehensive investigation of the DDR landscape can optimize glioma treatment.

**Methods:**

We identified the pronounced enrichment of DDR in TMZ-resistant glioma cells by RNA sequencing. Nine differentially expressed genes between TMZ-sensitive/resistant glioma cells were selected to construct the DDR score through lasso regression analysis. Two glioma cohorts from TCGA and CGGA were interrogated to evaluate the predictive ability of DDR score. Multiple algorithms were applied to estimate the immunotherapeutic responses of two DDR phenotypes. Immunohistochemistry was used to determine the protein levels of PD-L1 and TGFβ in glioma specimens. The oncoPredict package was employed to predict the candidate chemotherapy agents.

**Results:**

DDR score exhibited a robust prognostic capability in TCGA and CGGA cohorts and served as an independent predictive biomarker in glioma patients. Functional enrichment analyses revealed that high and low DDR score groups were characterized by distinct immune activity and metabolic processes. Elevated levels of infiltrating immune cells (including CD8+ T cells, CD4+ T cells, and dendritic cells) were observed in the high DDR score glioma. Further, high DDR scores correlated with increased mutation burden, up-regulated immune checkpoints, and tumor immunity activation, indicating a profound interplay between DDR score and glioma immunogenicity. In addition, PD-L1 and TGFβ were overexpressed in recurrent glioma specimens compared with primary ones. Finally, we estimated that PI3K inhibitors may serve as latent regimens for high DDR score patients.

**Conclusion:**

Our study highlighted the promising prognostic role of DDR score in glioma. Individual assessment of DDR status for patients with glioma may provide new clues for developing immunotherapeutic strategies.

## Introduction

DNA is under the continuous threat of various endogenous and exogenous stress, and efficient DNA damage response (DDR) and DNA repair are essential to maintain genomic integrity. DDR plays a critical role in regulating cell cycle, chromatin remodeling, cell metabolism, and apoptosis, and the deficiencies of DDR are usually associated with genomic instability and tumor initiation (1, 2). Interestingly, DDR defects also make tumor cells vulnerable to chemotherapy and radiotherapy because the damages caused by treatment cannot be effectively corrected. Thus, proficient DDR significantly usually contributes to cancer therapy resistance (3). O^6^-alkylguanine-DNA methyltransferase (MGMT) is a DNA repair enzyme that functions by transferring methyl groups from the O^6^ position of impaired guanine to its cysteine residues, accordingly blunting the efficacy of alkylating agents such as Temozolomide (TMZ) (4). TMZ chemotherapy is used as a first-line treatment in patients with glioma, however, the overall survival remains poor and the acquired chemoresistance induced by DDR is a major obstacle yet to be overcome (5). Therefore, there is a clear need to develop innovative therapeutic strategies and prognostic biomarkers.

Immune checkpoint blockade (ICB) therapy has made substantial breakthroughs and consolidated our understanding of immuno-oncology (6). Nevertheless, its clinical trials in glioma remain a formidable challenge largely due to the unique immune-privileged microenvironment. Glioma is surrounded by relatively low levels of pre-existing immune infiltration especially T cells, which is labeled as an immunologically cold phenotype (5, 7). Individual evaluation of glioma immunogenicity is therefore a prospective method to optimize patient selection and facilitate precise immunotherapy. In recent years, DDR deficiency has emerged as a predictive biomarker of response to ICB therapy in multiple cancers (8, 9). Tumors with mismatch repair (MMR) - deficiency (MMRd) has shown promising sensitivity to PD-1 blockade and consequently and FDA approved the application of pembrolizumab in patients with MMRd solid tumors. In addition, encouraging ICB sensitivity has been observed in tumors with other DDR defects including BRCA and POLE mutations (9). DDR-targeted treatments are considered to promote tumor immunogenicity by boosting antigenicity through accumulated tumor neoantigen burden (TNB), promoting adjuvanticity through the cytosolic immunity activation, and enhancing reactogenicity through the induction of immune checkpoints. In addition, DDR alterations have been reported to remodel the glioma immunosuppressive microenvironment by modulating M2 polarization of microglia (10, 11). But disappointingly, initial results of clinical trials revealed that gliomas with MMRd were characterized by the absence of prominent T cell infiltration, decreased patient lifespan, and a poor response rate to anti-PD1 therapy (12). Thus, we aim to comprehensively evaluate the DDR landscape and illuminate the interaction between DDR and immunogenicity in glioma.

Here, we established a DDR score system to predict the clinical outcome of glioma patients. Gliomas in different DDR score phenotypes displayed distinguished tumor microenvironment (TME) features and tumor immunogenicity, indicating that DDR evaluation potentially promote precise immunotherapy of glioma.

## Materials and Methods

### RNA Extraction and Sequencing

Total RNA was extracted from TMZ-sensitive/resistant U87MG cell lines by TRIzol reagent (Thermo Fisher Scientific, Waltham, MA, USA) following the manufacturer’s instructions. RNA quality was assessed by Nanodrop2000 and Qubit 3.0. RNA integrity was determined by Agilent 2100 Bioanalyzer. mRNA Capture Beads (Vazyme Biotech, Nanjing, China) were used to eliminate rRNAs and a VAHTS Total RNA-Seq Library Preparation Kit (Vazyme Biotech) was used to prepare libraries. Sequencing was performed on the Illumina Hiseq 2500 platform (pair-end 150 bp). The raw sequencing data have been deposited in the NCBI (BioProject accession: PRJNA768121). Data were further processed by R (version 4.1.0). A heatmap of gene expression profiles was generated using the pheatmap package. Principal component analysis (PCA) of each sample was performed and the top two principal components were shown. Differentially expressed genes (DEGs) were identified using the limma package (| log2(fold change) | > 1 and adjusted p-value < 0.05 as threshold). A volcano plot was illustrated to visualize the distribution of DDR genes using the EnhancedVolcano package.

### Glioma Data Acquisition

Two independent datasets of glioma patients were collected from the publicly available The Cancer Genome Atlas (TCGA) (https://xenabrowser.net/datapages) and Chinese Glioma Genome Atlas (CGGA) (http://www.cgga.org.cn) (13). TCGA and CGGA cohorts enrolled 557 and 656 glioma specimens, respectively. Patients lacking complete clinical annotations were excluded from subsequent analyses. Gene expression data from both cohorts were log2(FPKM+1) transformed.

### Construction of DDR Score

Univariate cox regression analysis was performed to screen the DDR-related DEGs between TMZ sensitive and resistant glioma cells. DEGs with significance in univariate cox regression were subsequently analyzed by LASSO regression to further select variables. To improve the accuracy of the risk model, LASSO regression was implemented with 10-fold cross validation and run for 1000 rounds to alleviate overfitting effects. Finally, nine DDR genes were picked out to construct the DDR score following the formula: $\text{DDR score}=\sum_{1}^{n}\text{Coef }\left(\text{i}\right)*\exp\left(i\right)$ , with Coef (i) meaning the coefficients of each variable and exp (*i*) representing the expression level of genes.

### Survival Analysis of DDR Score and Clinicopathological Factors

Glioma patients were divided into high and low DDR score groups according to the cutoff value. Scatter diagrams and Kaplan-Meier curves were generated by the survival package to evaluate the difference in clinical outcome between two DDR score phenotypes. ROC curves were depicted to determine the predictive capability of DDR score using the timeROC package. DDR scores of different glioma grades and pathological statuses were displayed using the ggplot2 package. Univariate and multivariate cox analyses were performed to investigate the prognostic value of DDR score and other clinicopathological indicators. The forest plot delineated the corresponding hazard ratio (HR) and p-value. A nomogram was constructed based on DDR score and other independent prognostic indicators to predict the 1, 2, and 3 years survival probability of glioma patients. Calibration curves were produced to evaluate the utility of the nomogram.

### Functional Annotation and Pathway Enrichment

DEGs between high and low DDR score groups were identified using the limma package (| log2(fold change) | > 1 and adjusted p-value < 0.05 as threshold). Symbols of DEGs were extracted using the clusterProfiler package to explore Gene Ontology (GO) and KEGG terms enrichment (14). Gene set enrichment analysis (GSEA) was used to investigate the enriched gene sets based on the fold changes of all genes. The significances were ranked by normalized enrichment score (NES) and adjusted p-value. Gene set variation analysis (GSVA) is a non-parametric, unsupervised approach to estimating the variation of gene set enrichment based on expression profiling. KEGG and HALLMARK gene sets were downloaded from the Molecular Signatures Database (MSigDB) and signatures of tumor metabolism were collected from a published study (15). The GSVA package was exploited to quantify the pathway activity by calculating the GSVA score of each sample (16).

### Characterization of Immune Infiltration in the TME

Tumor purity is diluted by the non-tumor components in the TME including immune infiltrates, stromal cells, blood vessels, and extracellular matrix. The ESTIMATE package was applied to calculate the immune score, stromal score, and tumor purity of glioma specimens (17). CIBERSORT was used to evaluate the proportions of 22 immune cell types based on deconvolution methods (18). ssGSEA was employed to investigate the levels of 28 immune cell types based on the marker gene signature score. TIMER was utilized to estimate the percentages of six immune cell types by linear least square regression (19). MCP-counter was implemented to quantify the absolute abundance of eight immune and two stromal cell populations based on the mean level of marker gene expression (20). Gene expression data with standard symbol annotation were imputed to the algorithms above for further analyses.

### Ethical Statement

Glioma specimens were obtained from patients who underwent surgical resection in Shanghai East Hospital (from 2019 to 2021). All participants signed written informed consent for molecular studies before sample collection. The clinical data of patients were recorded with the approval of the Ethical Committee and Institutional Review Board of Shanghai East Hospital.

### Immunohistochemistry Staining

Eight glioma samples were fixed by immersion in 10% formalin solution and then embedded in paraffin. 10-µm thick tissue sections were deparaffinized and rehydrated (xylene × 2 for 10 minutes each, 100%, 95%, and 75% ethanol for 5 minutes each and deionized water for 5 minutes). The sections were incubated with 3% hydrogen peroxide in methanol for 10 minutes to quench peroxidase activity. Antigen retrieval was performed by boiling sections in 10 mM sodium citrate buffer (pH 6.0) for 10 minutes. After being rinsed with PBS, sections were blocked with normal goat serum for 20 minutes. The samples were incubated with primary anti-PD-L1 (1:200, ab237726, Abcam, Cambridge, UK) or anti-TGFβ antibody (1:200, BA0290, BOSTER, Wuhan, China) overnight at 4°C. The sections were then incubated with secondary antibody () for 30 minutes at room temperature. The staining was developed using 3,3’-diaminobenzidine (DAB) as substrate and counterstained with hematoxylin. The sections were developed using 3,3’-diaminobenzidine (DAB) as substrate and counter-stained with Mayer’s Hematoxylin. All sections were independently reviewed by two pathologists according to the WHO criteria.

### Analysis and Visualization of Mutation Landscape

Somatic mutation files (SNPs and small INDELs) of TCGA glioma were downloaded from the UCSC Xena browser. The maftools package was used to present the mutational patterns of glioma specimens by the oncoplot function. The mutual exclusivity and co-occurrence of top frequent mutations were delineated by the somaticInteractions function. The mutation load of each specimen was calculated by the tmb function.

### Prediction of the Potential Chemotherapeutic Agents

Genomics of Drug Sensitivity in Cancer (GDSC) is a public dataset containing information on drug sensitivity in cancer cells and molecular markers of drug response (21). Using the oncoPredict package, GDSC2 gene expression profile and corresponding drug response information were downloaded to generate a ridge regression model that can be applied to glioma transcriptomic data (22). Then the sensitivity scores were yielded to predict the half-maximal inhibitory concentration (IC50) of all drugs in glioma patients.

### Immunotherapy Cohort

IMvigor210 is a cohort of 348 urothelial cancer patients treated with PD-L1 blockade therapy (23). The gene expression profiles, tumor mutation burden, neoantigen information, therapeutic responses, and survival data were downloaded using the IOBR package (24). Tumor-intrinsic signatures were derived from the IOBR package and enrichment scores were calculated by ssGSEA algorithm.

### Statistical Analysis

Statistical significance of normally distributed variables between two groups was analyzed by unpaired Student’s t-test and nonnormally distributed variables were examined by Wilcoxon test. The Kaplan–Meier survival curve was graphed to explore survival distributions. Log-rank test was used to determine statistical significance between groups. The Pearson correlation analysis was used to test the association between continuous variables. χ^2^-test was employed to analyze contingency tables. The multi-omics data were standardized by z-score scaling. All statistical analyses were conducted using R (version 4.1.0) and two-sided p-values less than 0.05 were considered statistically significant and labeled as *, p < 0.05; **, p < 0.01; ***, p < 0.001; and ****, p < 0.0001.

## Results

### DDR Was Remarkably Enriched in TMZ-Resistant Glioma Cells

**Figure 1:**
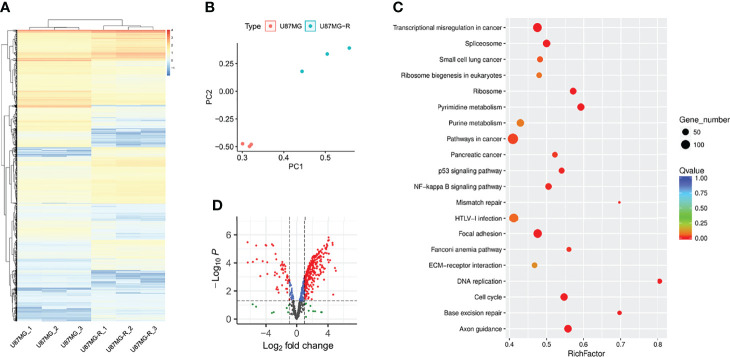
DDR was enriched in TMZ-resistant glioma cells. **(A)** The heatmap presented the gene expression profiling of U87-MG and U87-MGR cells. **(B)** PCA distinguished the U87-MG and U87-MGR cells. **(C)** The differentially enriched KEGG pathways between U87-MG and U87-MGR cells. **(D)** The volcano plot showed the distribution of DDR-related DEGs between U87-MG and U87-MGR cells.

To investigate the underlying mechanisms of TMZ resistance in glioma cells, we used RNA sequencing to analyze the transcriptomic alternations in TMZ-resistant U87-MG (U87-MGR) cells. U87-MG and U87-MGR cells exhibited distinct transcriptomic traits and principal components (**Figures 1A, B**). KEGG analysis revealed that DDR pathways were differentially enriched between TMZ sensitive and resistant cells including DNA replication, base excision repair (BER), MMR, Fanconi anemia, and cell cycle (**Figure 1C**). We gathered a list of 608 genes regulating DNA replication and/or DNA repair from Molecular Signatures Database (MSigDB) to screen out the DDR-related DEGs. The overall distribution was shown in a volcano plot and the red dots represented the DEGs (**Figure 1D**).

### DDR Score Was Constructed to Investigate the Predictive Value in Glioma

**Figure 2:**
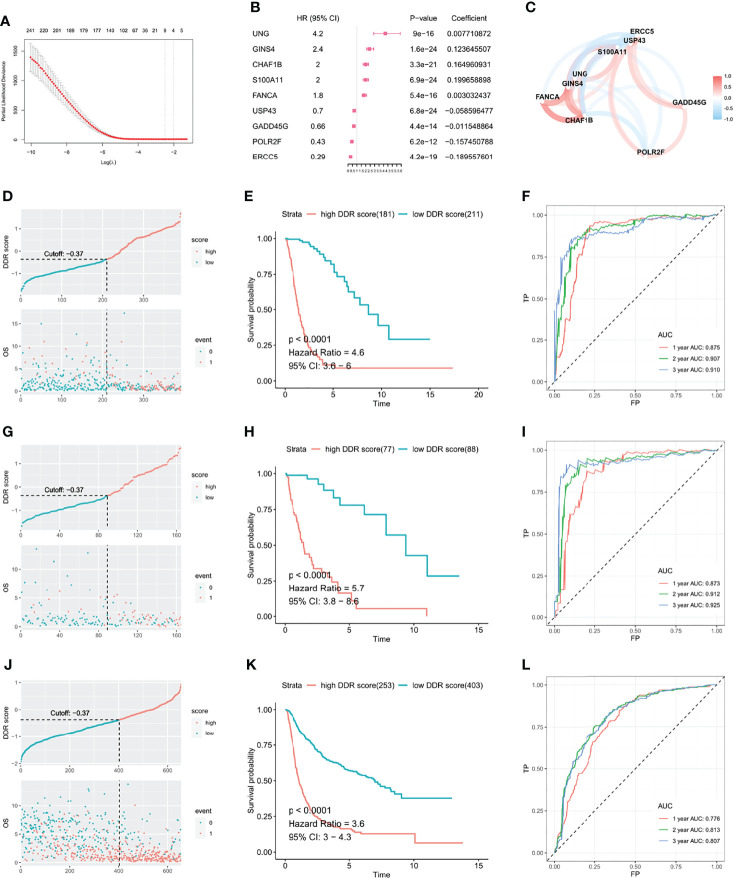
DDR score was established to investigate the predictive value in glioma. **(A)** DDR-related DEGs were further selected by LASSO cox regression to generate a risk model. **(B)** Coefficients and HR calculated by LASSO cox regression of nine variates. **(C)** The mutual relationship of the expression level of the nine genes. The scatter plot and Kaplan-Meier curves demonstrated that the high DDR score correlated with an unfavorable outcome in the TCGA training cohort **(D, E)**, TCGA validation cohort **(G, H)**, and CGGA validation cohort **(J, K)**. ROC curves suggested that the DDR score substantially predicted the prognosis in the TCGA training cohort **(F)**, TCGA validation cohort **(I)**, and CGGA validation cohort **(L)**.

To establish a DDR risk model, we selected nine genes from the DDR-related DEGs using univariate cox regression and LASSO regression analyses (**Figure 2A** and **Supplementary Figure 1A**). We constructed the DDR score based on the expression levels of nine genes and corresponding coefficients (**Figure 2B**): DDR score = exp (UNG) × 0.007710872 + exp (GINS4) × 0.123645507 + exp (CHAF1B) × 0.164960931 + exp (S100A11) × 0.199658898 + exp (FANCA) × 0.003032437 + exp (USP43) × -0.058596477 + exp (GADD45G) × -0.011548864 + exp (POLR2F) × -0.157450788 + exp (ERCC5) × -0.189557601. A network plot showed the mutual relationship of these nine genes (**Figure 2C**). An optimized cut-off DDR score of -0.37 was used for classification into high and low DDR score glioma patients. The scatter plot and Kaplan-Meier curves revealed that a high DDR score was associated with a poorer outcome in the TCGA training cohort (**Figures 2D, E**). ROC analysis indicated that the area under the curve (AUC) for 1, 2, and 3 years survival were 0.875, 0.907, and 0.910, respectively (**Figure 2F**). Similar survival analysis results were observed in the TCGA validation cohort (**Figures 2G, H**) and the corresponding AUC values were 0.873, 0.912, and 0.925 (**Figure 2I**). Furthermore, the AUC for 1, 2, and 3 years survival were 0.872, 0.906, and 0.912 in the entire TCGA cohort (**Supplementary Figure 1B**). Then we applied CGGA data for external validation, patients in the high DDR score group also had disadvantageous survival compared with counterparts (**Figures 2J, K**). The AUC values for 1, 2, and 3 years survival were 0.776, 0.813, and 0.807, respectively (**Figure 2L**). Taken together, survival analysis underlined the robust value of DDR score for predicting glioma prognosis.

### DDR Score Was an Independent Prognostic Factor Among Other Clinical Parameters

**Figure 3:**
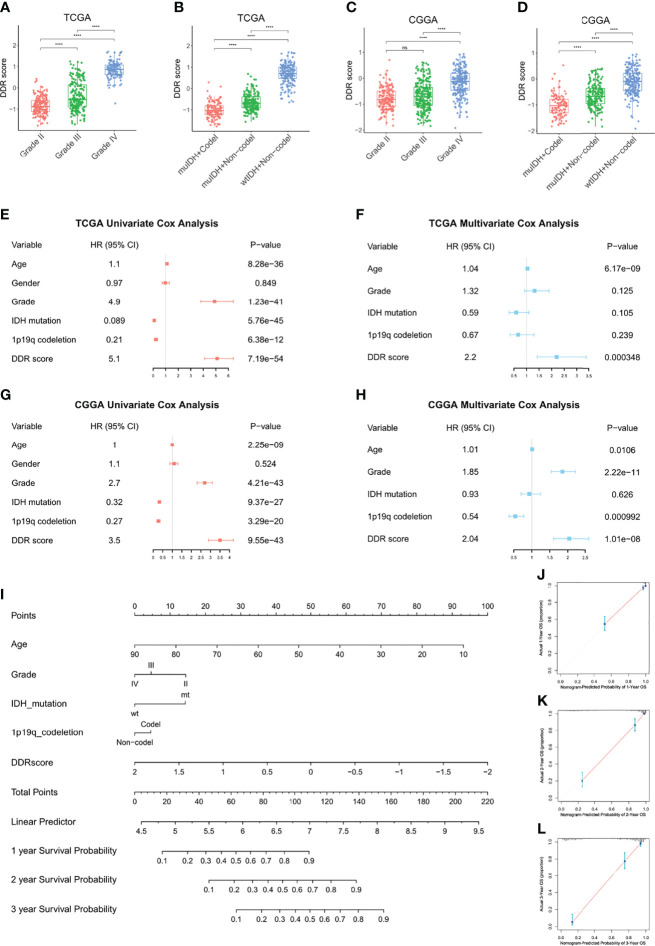
DDR score was an independent prognostic factor among other clinical parameters. **(A–D)** The DDR scores were compared between patients with different malignancy grades and clinicopathological statuses in TCGA **(A, B)** and CGGA **(C, D)**. **(E–H)** Univariate and multivariate cox analyses of DDR score, tumor grade, IDH mutation status, and 1p19q codeletion status in the TCGA **(E, F)** and CGGA cohorts **(G, H)**. **(I)** A nomogram to predict the 1, 2, and 3 years survival probability of the TCGA cohort. **(J–L)** The calibration curves of the nomogram to predict the 1, 2, and 3 years survival probability. ****p < 0.0001; ns, no significance.

Then we assessed the DDR score of patients with different malignancy grades and pathological statuses in TCGA and CGGA. High grade (grade IV) tumors exhibited higher DDR scores than low grade (grade II and III) ones, and gliomas with IDH wildtype and 1p19q non-deletion showed the highest DDR score compared with other clinicopathological subtypes (**Figures 3A–D**). Moreover, the Kaplan-Meier curves revealed that high DDR score group showed significantly shorter lifespan and worse outcome in IDH mutant lower-grade gliomas (**Supplementary Figures 2A, B**). We evaluated the prognostic value of DDR score and clinicopathological parameters in TCGA using univariate cox regression analysis. The HR and p-value of each factor were shown in a forest plot (**Figure 3E**). Variables with a significant p-value were taken into a multivariate model and the results indicated that DDR score and age were independent prognostic factors in the TCGA cohort (**Figure 3F**). Univariate and multivariate cox analyses also revealed that DDR score, tumor grade, and 1p19q codeletion status independently predicted the prognosis of patients in the CGGA cohort (**Figures 3G, H**). In addition, a nomogram was constructed by integrating the DDR score and other parameters to predict the 1, 2, and 3 years survival probability of glioma patients in TCGA (**Figure 3I**). The corresponding calibration curves were close to the ideal model (**Figures 3J–L**). The nomogram and calibration curves of the CGGA cohort were present in **Supplementary Figures 2C–F**.

### Functional Annotations and Pathway Enrichment Analyses of DDR Score Subtypes

**Figure 4:**
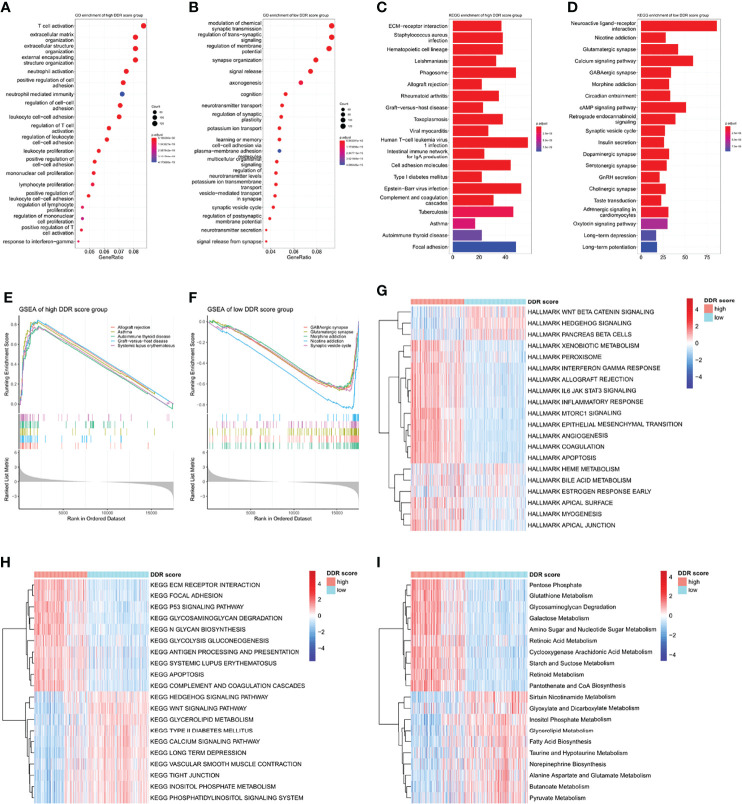
Functional annotations and pathway enrichment analyses of DDR score subtypes **(A, B)** GO annotations of the high and low DDR score groups. **(C, D)** KEGG analysis of the high and low DDR score groups. **(E, F)** GSEA of the high and low DDR score groups. **(G, H)** HALLMARK and KEGG signature enrichment of high and low DDR score subtypes calculated by the ssGSEA algorithm. **(I)** The differentially enriched metabolic processes in high and low DDR score patients.

To understand the underlying biology contributing to the extraordinary predictive ability of the DDR score, we explored the GO enrichment in TCGA using the clusterProfiler package. T cell activation was remarkably connected to a high DDR score while signal transduction-related genes were overexpressed in the low DDR score subset (**Figures 4A, B**). KEGG and GSEA analyses revealed that pathways involved in immune system disorders were significantly associated with high DDR score and synaptic transmission pathways were enriched in the low DDR score group (**Figures 4C–F**). Then we comprehensively investigated the HALLMARK and KEGG signature scores of each sample using the ssGSEA algorithm. The significances were ranked by adjusted p-values using the limma algorithm and the top 20 enrichment were visualized in heatmaps (**Figures 4G, H**). High DDR score gliomas exhibited the enrichment of multiple immune activation pathways including IL-6/JAK/STAT3 pathway, interferon-gamma response, inflammatory response, and antigen processing and presentation. Low DDR score correlated with the enrichment of the Wnt signaling pathway and Hedgehog signaling pathway. In addition, we observed the different metabolic regulations between two phenotypes so we further explored the potential metabolic mechanisms exploiting relevant signatures. The results underlined that cyclooxygenase arachidonic acid metabolism, glutathione metabolism, and pentose phosphate were upregulated in the high DDR score group, while alanine aspartate and glutamate metabolism, fatty acid biosynthesis, and sirtuin nicotinamide metabolism were stimulated in the low DDR score phenotype (**Figure 4I**).

### Immune Infiltrating Patterns of DDR Score Phenotypes

**Figure 5:**
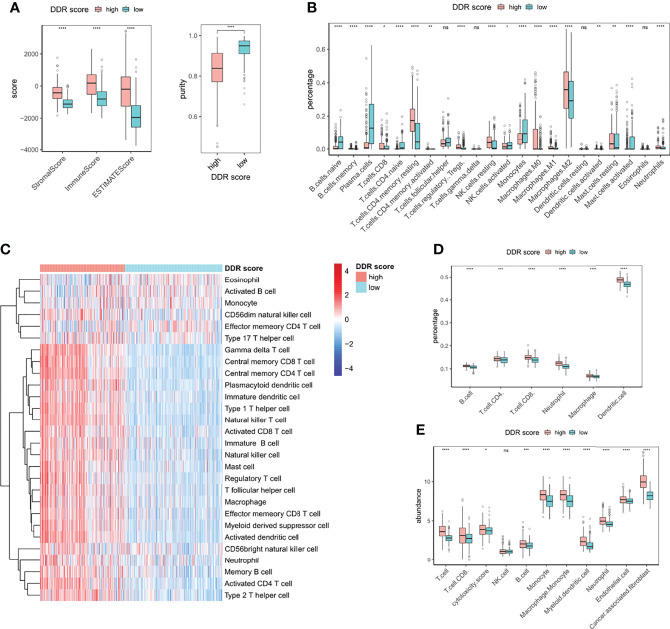
Immune infiltrating patterns of DDR score phenotypes. **(A)** Levels of stromal score, immune score, estimate score, and tumor purity in high and low DDR score groups. **(B)** The fractions of 22 types of immune cells in high and low DDR score groups based on CIBERSORT. **(C)** The infiltrating levels of 28 subpopulations of immune cells in high and low DDR score groups based on ssGSEA. **(D)** The percentages of six immune cell types in high and low DDR score groups based on TIMER. **(E)** The abundances of eight immune and two stromal cell populations in high and low DDR score groups based on MCP-counter. *p < 0.05; **p < 0.01; ***p < 0.001; ****p < 0.0001; ns, no significance.

The tumor is surrounded by a dynamic microenvironment that consists of various types of infiltrating immune cells. The interplay between these immune components and tumor cells can shape tumor immunogenicity and affect the tumor response to checkpoint inhibitors (6). Considering the distinct immune and metabolic characteristics of high and low DDR score subsets, we then investigated the association between DDR score and immune infiltration. The stromal score, immune score, and tumor purity of glioma specimens were inferred using the ESTIMATE package. High DDR score correlated with elevated stromal and immune scores but decreased glioma purity (**Figure 5A**), indicating the raised levels of stromal and immune cells in the TME of high DDR score glioma. We then further explored the TME landscape of two DDR score subtypes using CIBERSORT, ssGSEA, TIMER, and MCP-counter algorithms (**Figures 5B–E**). Tumors with high DDR scores correlated with elevated levels of multiple immune infiltration. Interestingly, we discovered that both pro-tumor (myeloid-derived suppressor cells (MDSCs), regulatory T cells (Tregs), M2 macrophages, and immature dendritic cells) and anti-tumor (CD8+ T cells, CD4+ T cells, activated dendritic cells, and M1 macrophages) immune cells were up-regulated. Notably, tumors with the presence of CD4+ and CD8+ T cells were defined as immune-inflamed and usually associated with a positive response to ICB. Activated dendritic cells and M1 macrophages are effective in T cell activation through antigen presentation. On the other hand, MDSCs can weaken the activity of effector T cells, mediate the differentiation of Tregs, and promote an immunosuppressive phenotype in macrophages. Immature dendritic cells induce an immunosuppressive TME through expanding Tregs. Further, M2 macrophages contribute to tumor immune evasion by expressing anti-inflammatory cytokines and attenuating the activity of CD8+ T cell (25). In a nutshell, the complicated TME of glioma was characterized by the mixture of pro- and anti-tumor cells, as well as the coexistence of immune activation and suppression.

### DDR-Associated Immune Microenvironment Characteristics

**Figure 6:**
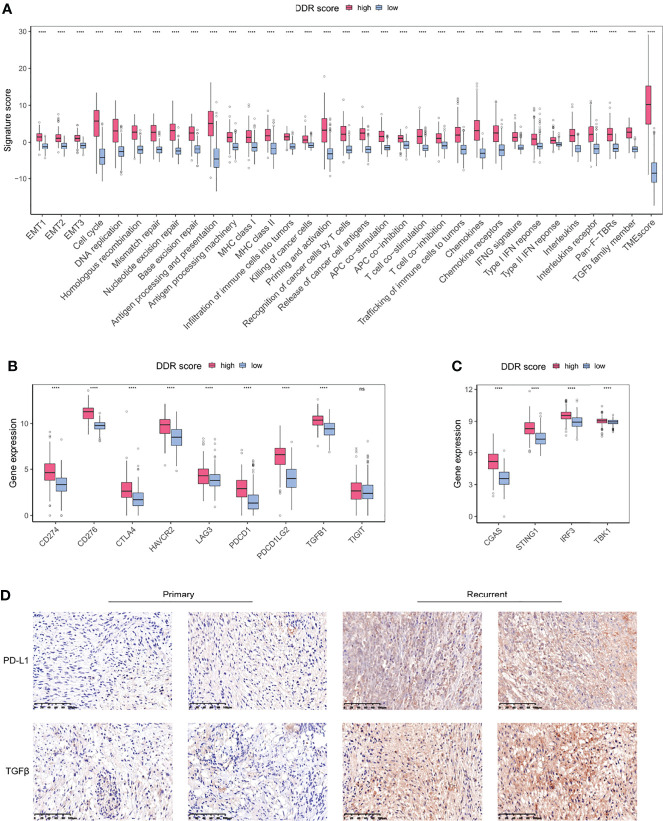
DDR-associated immune microenvironment signatures and characteristics. **(A)** The differentially expressed TME signatures in high and low DDR score groups. **(B)** The expression levels of immune checkpoints in high and low DDR score groups. **(C)** The expression levels of cGAS-STING pathway members in high and low DDR score groups. **(D)** IHC staining of PD-L1 and TGFβ in primary and recurrent glioma tissues (20×magnification). EMT, epithelial-to-mesenchymal transition; MHC, major histocompatibility complex; HLA, human leukocyte antigen; IFN, interferon; Pan-F-TBR, pan-fibroblast TGFβ response. ****p < 0.0001.

Given the differential infiltrating immune cells in two DDR groups, we sought to explore the signatures of TME associated with DDR score. All the defined gene signatures were obtained from previous publications (23, 26–30). DDR-related processes were overexpressed in high DDR score specimens, indicating an overexpressed DDR phenotype (**Figure 6A**). Antigen processing, chemokines, and interferon responses were significantly up-regulated in the high DDR score group, suggesting the enhanced efficiency for T cells to recognize antigens and the triggered inflammation and antitumor immunity. TMEscore was a novel biomarker with high sensitivity in predicting immunotherapy efficacy, and we identified its positive correlation with DDR score. Intriguingly, high DDR score gliomas also showed promoted TGFβ pathway activity which was associated with immunosuppression (**Figures 6A, B**). These observations revealed the coexistence of anticancer immunity activation and immune suppression in the glioma microenvironment.

Immune checkpoints are regulators of immunological tolerance that function to protect the cells from indiscriminate attack. The activation of inhibitory checkpoint molecules prevents tumors from damage and attack so they can serve as promising targets for cancer immunotherapy (31). We investigated the immune checkpoint expression in glioma specimens and uncovered that CD274 (PD-L1), PDCD1, PDCD1LG2, CTLA4, CD276, HAVCR2, and LAG3 were significantly overexpressed in the high DDR score subtype (**Figure 6B**). The cGAS-STING pathway is an important component of the cellular innate immune system that functions by detecting cytosolic DNA fragments and consequently triggers cytosolic immunity. Stimulating cytosolic immunity is a state-of-the-art strategy to optimize ICB therapy efficacy by promoting infiltrating T cells to turn immunologically cold tumors into hot tumors (32). Our analysis suggested that the high DDR score subset presented the elevated levels of cGAS-STING pathway members (**Figure 6C**). Using immunohistochemistry (IHC) analysis, we evaluated the protein expression of PD-L1 and TGFβ in primary and recurrent gliomas. Recurrent gliomas, usually concomitant with chemotherapy and/or radiotherapy resistance, were considered to be in a DDR proficiency status. IHC staining showed that PD-L1 and TGFβ were up-regulated in recurrent tumors compared to counterparts (**Figure 6D**). Collectively, we identified a collection of differentially expressed TME gene signatures, which potentially indicated an encouraging sensitivity of ICB in high DDR score gliomas.

### Correlation of DDR Score and Glioma Somatic Genome

**Figure 7:**
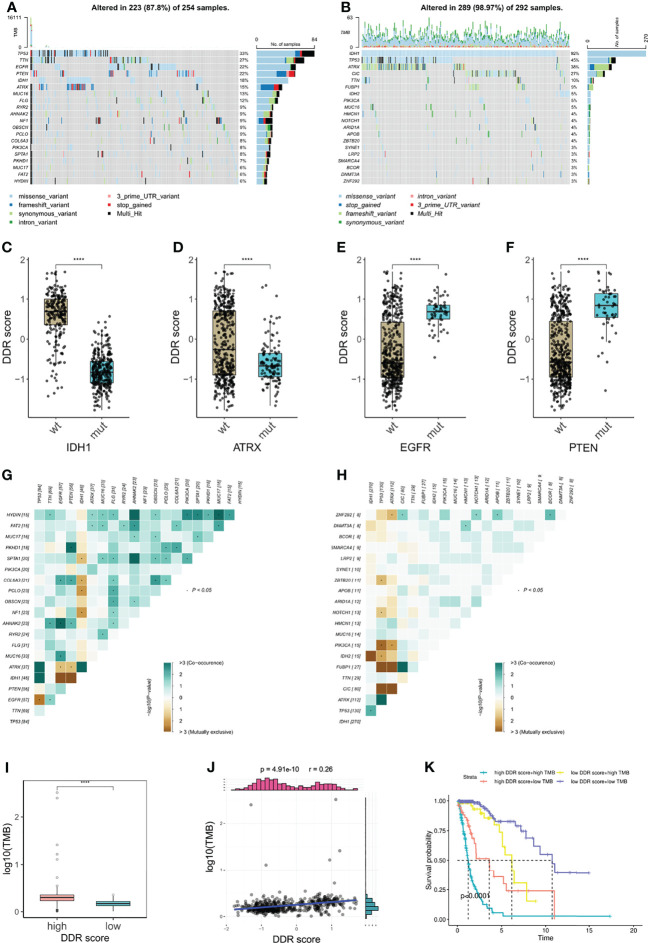
Correlation of DDR score and glioma somatic genome. **(A, B)** The top 20 frequent somatic mutations in high and low DDR score groups. **(C–F)** IDH1, ATRX, EGFR, and PTEN mutations were significantly correlated with DDR score levels. **(G, H)** Mutual exclusivity and co-occurrence of mutations in high and low DDR score groups. **(I)** TMB level in high and low DDR score groups. **(J)** The correlation of DDR score and TMB. **(K)** The Kaplan-Meier survival analysis for patients stratified by both DDR score and TMB. ****p < 0.0001.

Tumor mutation burden (TMB) is defined as the number of genetic mutations in a tumor. High tumor mutation burden usually correlates with a positive response to ICB therapy substantially because increasing mutation load potentially generates neoantigens to enhance tumor antigenicity and immunogenicity (33). To outline the somatic mutation landscape of two DDR subtypes, we displayed the top 20 frequent mutations in both groups, (**Figures 7A, B**). 18% of high DDR score patients exhibited IDH1 mutation (**Figure 7A**) but conspicuously the alternation rate of IDH1 was up to 92% in low DDR score phenotype (**Figure 7B**). Therefore, DDR score seemed to be a robust indicator of IDH1 mutation status in glioma. Further, DDR scores of IDH1 mutant patients were notably lower than that of IDH1 wildtype patients (**Figure 7C**), which was consistent with our results in **Figures 3B, D**. Meanwhile, ATRX mutant gliomas also showed significantly decreased DDR score (**Figure 7D**). Conversely, EGFR and PTEN mutations were crucially correlated with the increase of DDR score (**Figures 7E, F**). These results suggested that EGFR and PTEN mutations may potentiate DDR activity in glioma.

Additionally, higher frequencies of mutation co-occurrences were observed in high DDR score gliomas (**Figures 7G, H**). Subsequently, we calculated the TMB of each glioma specimen and identified that the DDR score was positively associated with the mutation burden (**Figures 7I, J**). Nevertheless, no such correlation was observed in IDH wildtype glioblastoma (GBM) (**Supplementary Figure 3**). Based on the median TMB and the DDR score cut-off value, we divided patients into four groups and uncovered that patients with high DDR scores and high TMB associated with the worst prognosis and those with low DDR scores and low TMB showed the longest survival (**Figure 7K**).

### DDR Score Subtypes Guided Chemotherapy Strategies

**Figure 8:**
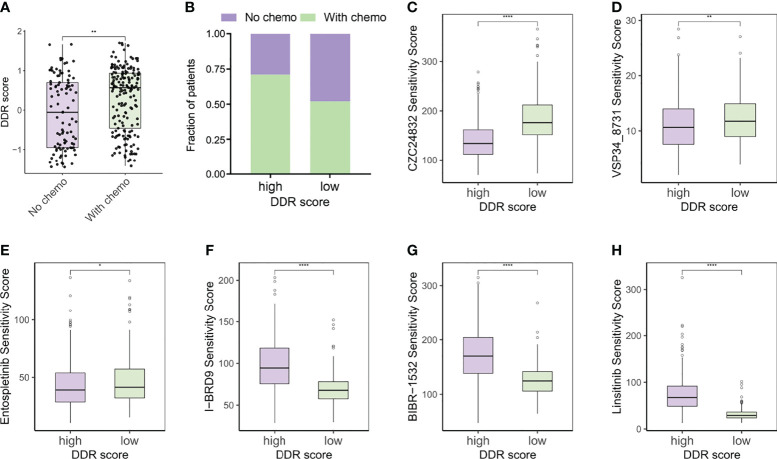
DDR score subtypes guided chemotherapy strategies. **(A)** The DDR scores of patients with or without chemotherapy. **(B)** Rate of chemotherapy statuses (No/With chemo) in high and low DDR score groups. **(C–E)** Predicted sensitivity scores of CZC24832, VSP34_8731, and Entospletinib, which were candidate chemotherapeutic agents for high DDR score patients. **(F–H)** Predicted sensitivity scores of I-BRD9, BIBR-1532, and Linsitinib, which were candidate potent drug options for low DDR score patients. *p < 0.05; **p < 0.01; ****p < 0.0001.

Immunogenic cell death prompted by certain chemotherapy agents can be exploited to sensitize tumors to checkpoint blockade, so the optimal combination of chemotherapy and immunotherapy warrants further exploration (34, 35). Since the DDR score was generated based on the DEGs of TMZ-resistant glioma cells, we supposed that chemotherapy status potentially correlated with DDR score levels. Our analysis revealed that DDR scores were higher in patients who underwent chemotherapy (**Figures 8A, B**). OncoPredict package was utilized to predict the sensitivity scores of drugs in high and low DDR score groups and the sensitivity score was positively correlated with the IC50 value of chemotherapy agents. We compared the estimated TMZ sensitivity between two subtypes and found no significance (**Supplementary Figure 4A**). In addition, MGMT and BCL3 were up-regulated in the high DDR score group but no statistical significance of ALKBH2 expression was observed (**Supplementary Figure 4B**). Therefore, more studies were needed to investigate the correlation between DDR score and the susceptibility of alkylating agents. Further analysis suggested that targeting the PI3K pathway (CZC24832 and VSP34_8731) and inducing apoptosis (Entospletinib) may be efficient strategies for high DDR score patients (**Figures 8C–E**). These predictions were hardly surprising because apoptosis and mTORC1 signaling were enriched in the high DDR score phenotype according to the aforementioned functional analysis. Meanwhile, I-BRD9 (BRD9 inhibitor), BIBR-1532 (telomerase inhibitor), and Linsitinib (IGF-1R inhibitor) were candidate drugs for the treatment of low DDR score tumors (**Figures 8F–H**).

### Validation of DDR Score in a Checkpoint Immunotherapy Cohort

A growing body of early-phase clinical trials have been developed to evaluate the potential of combining DDR-targeted therapy with ICB (9). Currently, most reports regarding DDR-immunity interaction are focused on DDR deficiency including MMRd, homologous recombination - deficiency (HRD), and deleterious DDR mutations (23, 36). However, the role of overexpressed DDR profiles has been hardly investigated in immuno-oncology. We calculated the tumor-intrinsic signature scores of the urothelial cancer patients from a PD-L1 blockade cohort and the analysis indicated that DDR-related signatures were remarkably up-regulated in immunotherapy responders (**Supplementary Figure 5A**). Patients in response to anti-PD-L1 therapy exhibited higher signature scores of cell cycle, MMR, and homologous recombination signatures. Further, favorable therapy responses also correlated with increased DDR scores (**Supplementary Figure 5B**). Then the samples were divided into high and low DDR score groups according to the median score value. Intriguingly, high DDR score patients showed elevated TMB and TNB levels and prolonged survival (**Supplementary Figures 5C–E**). In summary, DDR score may predict the sensitivity of checkpoint immunotherapy in certain cancer types.

## Discussion

DDR deficiency is closely connected to genomic instability and tumorigenesis, but in contrast, DDR also confers resistance to anticancer agents in various tumors (3). Unmethylated MGMT promoter creates a resistant glioma phenotype by restoring the DNA alkylation and serves as an essential contributor to chemotherapy failure (4). Recently, the DDR-targeted strategy has opened new therapeutic avenues for antitumor immunity and some DDR-related biomarkers have exhibited reliable predictive capability in ICB therapy (9). Therefore, it is plausible to assume that comprehensively evaluating the DDR status can optimize therapeutic effects and develop combinatorial treatment strategies.

In this study, we constructed a DDR score system based on the TMZ-resistant signature in glioma cells. DDR score exhibited great prognostic value and independently predicted the survival of glioma patients. High DDR score correlated with enhanced antigenicity by increasing mutation burden and activating antigen processing and presentation. Tumors with elevated TMB were more likely to generate neoantigens for triggering antitumor T cell responses. Moreover, lower DDR scores were observed in gliomas with IDH1 and ATRX mutations, but on the other hand, EGFR and PTEN mutations were associated with high DDR scores. This was consistent with the findings suggesting that ATRX knockout can suppress DNA damage repair by regulating ATM pathway or mediating PARP1 instability to sensitize glioma cells to TMZ treatment (37, 38). Interestingly, a recent study demonstrated that ATRX loss promoted malignant and immunosuppressive phenotypes of IDH1 mutant glioma cells (39). Another study reported that non-small cell lung cancer patients with EGFR mutation and higher PD-L1 expression may benefit from PD-1 inhibitors (40).

Two DDR score subtypes also showed distinct TME landscapes. High DDR scores were directly proportional to the infiltration of multiple immune cells, suggesting the coexistence of pro- and anti-tumor constituents in the TME. Among them, pre-existing T cell infiltration has been closely related to antitumor immunity in patients with ICB therapy. Furthermore, GO annotation and metabolic analysis denoted the positive regulation of T cell activation and glutathione metabolism in the high DDR score group. Activated T cells can produce reactive oxygen species (ROS) to promote the antioxidative glutathione, thus priming T cell metabolism for inflammation (41). We also identified the pentose phosphate pathway (PPP) activation in the high DDR score phenotype. PPP is a major cellular source for NADPH, which is needed for fatty acid synthesis and redox homeostasis in early activated T cells and inflammatory macrophages (42). On the other hand, the high DDR score group also had a larger fraction of MDSCs and Tregs, which were usually associated with immune evasion in cancers. From the metabolic perspective, the enrichment of arachidonic acid metabolism in the high DDR score group has been reported to engage in the immunosuppressive function of MDSCs (43). In addition, other regulators of immunosuppression such as TGFβ and PD-L1 were up-regulated in the high DDR score gliomas. These results highlighted the complication of TME by the concomitant presence of immune activation and immune suppression. Nevertheless, the elevated levels of immune checkpoints can exactly make high DDR score patients benefit more from checkpoint inhibitors (31).

Of note, cytosolic immunity was activated in the high DDR score group by up-regulating the cGAS-STING pathway, interferon responses, and chemokines. Defective DDR-induced nucleic acid fragments can be detected by cGAS and consequently stimulate type I interferon responses and proinflammatory cytokines (32). It seemed to be a paradox when we observed triggered tumor innate immunity in the high DDR score group, which was a phenotype with DDR enrichment. Glioma patients with chemotherapy were proved to have high DDR scores so we supposed that continuous alkylating agent-induced damage contributed to the chronic activation of cytosolic immune responses. Additionally, chronic activation of the cGAS-STING pathway has been found to drive tumor metastasis and cancer progression (44), which may partly elucidate the high malignancy and poor survival of patients with increased DDR scores.

Combinational strategies of conventional chemotherapy with ICB are underway to tackle resistance and extend the application of immunotherapy. However, the combination may not be greater than the sum of its parts because many chemotherapies are considered to shape an immunosuppressive TME (35). Systemic TMZ therapy is well known to induce lymphopenia in glioma and the effects of TMZ on immune cells and TME are largely dependent on the timing and dosing regimen (45). Hence, the combinatorial treatments using TMZ and immunotherapy require thoughtful consideration. In our study, the computational analysis revealed that PI3K inhibitors may be potent options to treat high DDR score patients. Notably, previous publications suggest that PI3K inhibitors interfere with suppressive myeloid and macrophage features of the TME to overcome the therapeutic resistance to ICB (46, 47).

Certainly, our study still has some limitations. Due to the lack of a prospective cohort of glioma patients receiving ICB treatment, we explored the correlation of DDR score and anti-PD-L1 therapy sensitivity in a urothelial cancer cohort instead. Additionally, we used algorithm analyses to predict the prognostic value of DDR score in the public database but not verified it in our patient data so we hope to collect more specimens for multi-omics analysis in future validation.

## Conclusion

This study highlighted the promising prognostic value of DDR score in patients with glioma. A comprehensive assessment of DDR status in glioma may be conducive to developing individualized immunotherapy and guiding innovative drug combinatorial strategies.

## Data Availability Statement

The datasets presented in this study can be found in online repositories. The names of the repository/repositories and accession number(s) can be found below:

https://www.ncbi.nlm.nih.gov/bioproject/ PRJNA768121.

## Ethics Statement

The studies involving human participants were reviewed and approved by Committee and Institutional Review Board of Shanghai East Hospital. The patients/participants provided their written informed consent to participate in this study.

## Author Contributions

MChen prepared RNA sequencing, analyzed data, and illustrated figures. LZ, QW, and HL contributed to bioinformatics analysis. HB, YP, MCheng, and ML helped tissue collection and conducted IHC analysis. MChen, HB, LZ and QW wrote the manuscript. KZ participated in manuscript review and editing. CZ, JZ and SX conceived the study, supervised the work, and revised the manuscript. All authors contributed to the article and approved the submitted version.

## Funding

This research was supported by grants from the National Natural Science Foundation of China (82172820), the Fundamental Research for the Central University, the Natural Science Foundation of Shanghai (22ZR1466200, 22ZR1451200), the Outstanding Leaders Training Program of Pudong Health Bureau of Shanghai (PWR12018-07), the Medical Discipline Construction Project of Pudong Health Committee of Shanghai (PWYgy 2021-07), and the Key Discipline Construction Project of Pudong Health Bureau of Shanghai (PWZxk2017-23).

## Conflict of Interest

The authors declare that the research was conducted in the absence of any commercial or financial relationships that could be construed as a potential conflict of interest.

## Publisher’s Note

All claims expressed in this article are solely those of the authors and do not necessarily represent those of their affiliated organizations, or those of the publisher, the editors and the reviewers. Any product that may be evaluated in this article, or claim that may be made by its manufacturer, is not guaranteed or endorsed by the publisher.
