# Supplementary material for: DNA Damage Response Evaluation Provides Novel Insights for Personalized Immunotherapy in Glioma
Source: Front Immunol. 2022 May 26;13:875648. doi: 10.3389/fimmu.2022.875648 (PMC9204352; doi:10.3389/fimmu.2022.875648)
Supplement: Supplementary file 1 [file DataSheet_1.pdf]

Supplementary Materials

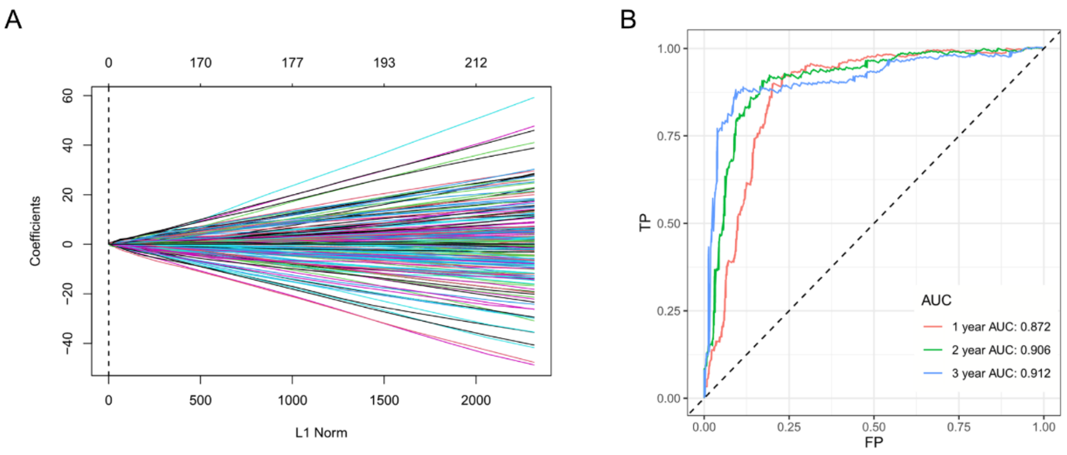

Supplementary Figure 1 (A) The profile plot for LASSO coefficients of the 9 variates. **(B)** The AUC for 1, 2, and 3 years survival were 0.872, 0.906, and 0.912 in the entire TCGA cohort.

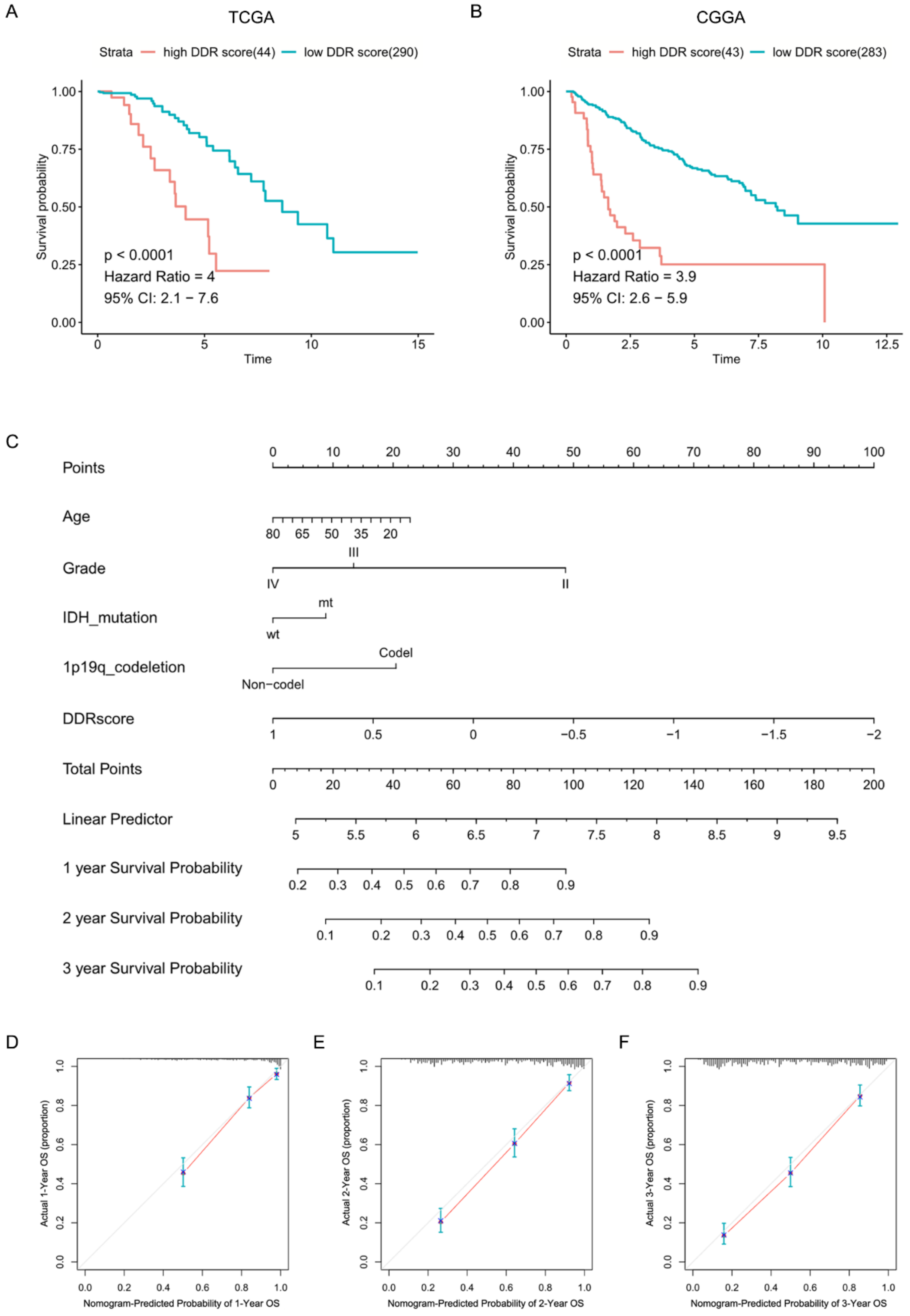

Supplementary Figure 2 (A, B) High DDR score group showed worse clinical outcome in IDH mutant lower-grade gliomas from TCGA (A) and CGGA (B). **(C)** A nomogram to predict the 1, 2, and 3 years survival probability of CGGA cohort. **(D-F)** The calibration curves of the nomogram to predict the 1, 2, and 3 years survival probability.

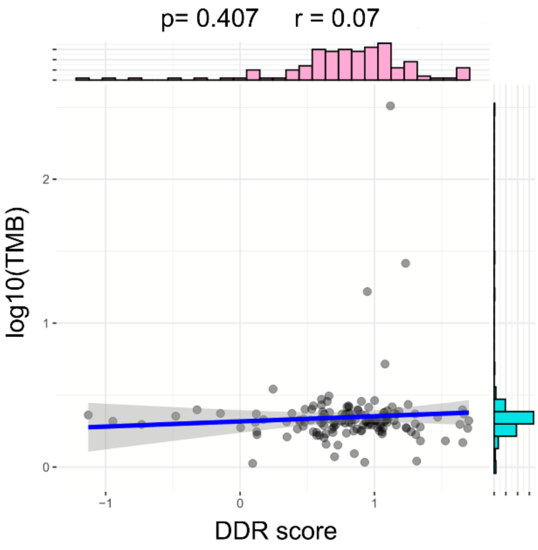

Supplementary Figure 3 No significant correlation between DDR score and TMB in IDH wildtype GBM.

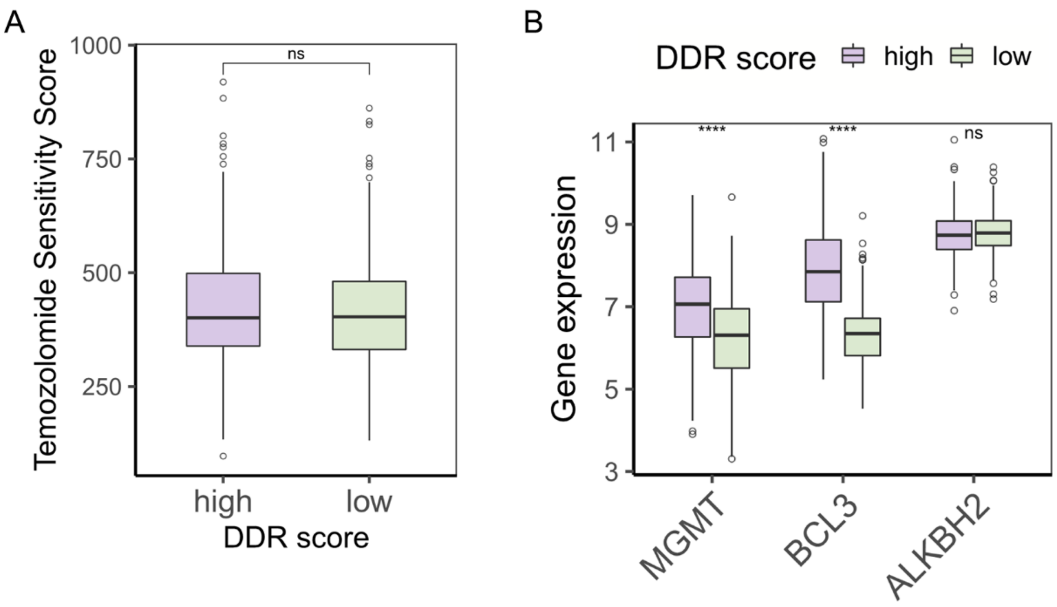

Supplementary Figure 4 (A) Predicted sensitivity score of TMZ in high and low DDR score groups. **(B)** Expression levels of MGMT, BCL3, and ALKBH2 in high and low DDR score groups.

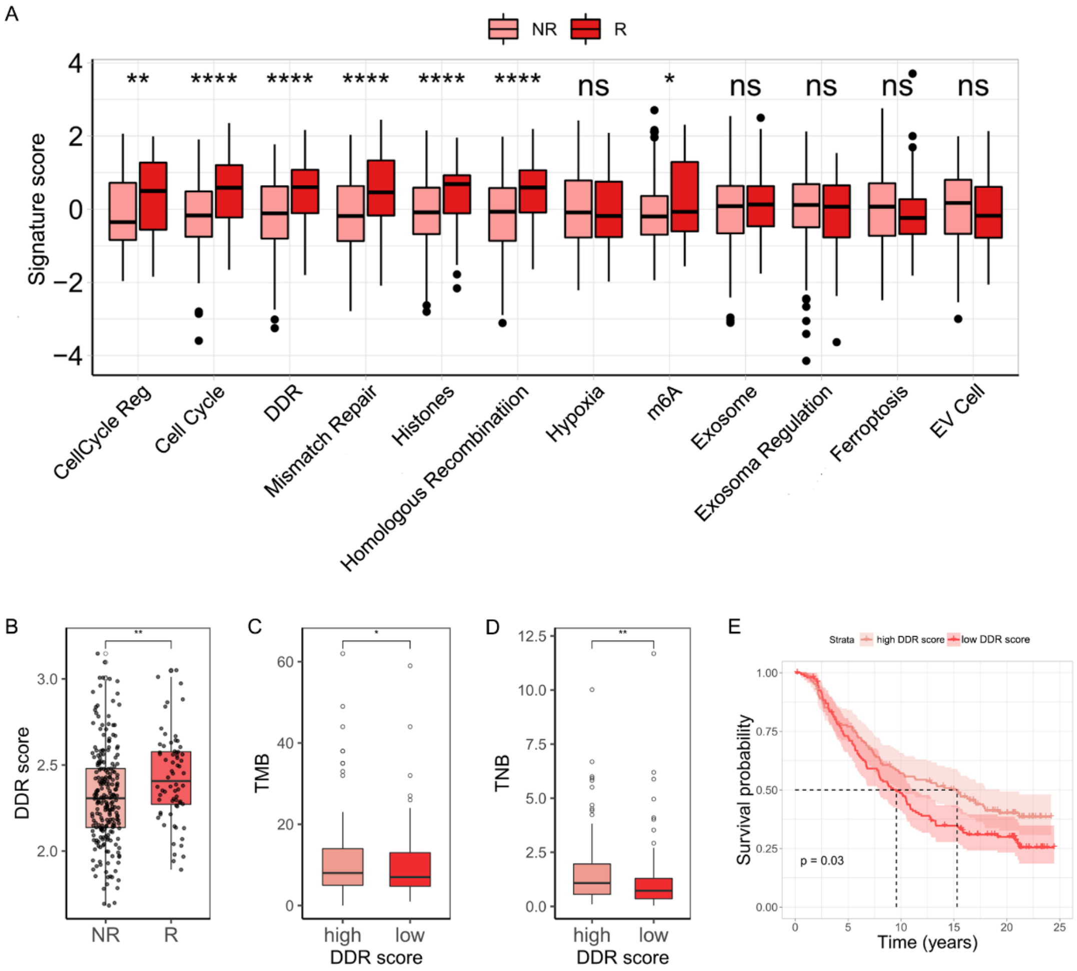

Supplementary Figure 5 (A) The tumor-intrinsic signature scores of the responders (R) and non-responders (NR) to PD-L1 blockade from a urothelial cancer cohort. **(B)** The DDR scores of R and NR to immunotherapy. **(C)** TMB levels in high and low DDR score groups. **(D)** TNB levels in high and low DDR score groups. **(E)** The Kaplan-Meier curves demonstrated that the high DDR score correlated with a favorable outcome in the immunotherapy cohort.
